# Morphology and ultrastructure of *Interfilum* and *Klebsormidium* (Klebsormidiales, Streptophyta) with special reference to cell division and thallus formation

**DOI:** 10.1080/09670262.2014.949308

**Published:** 2014-10-13

**Authors:** Tatiana Mikhailyuk, Andreas Holzinger, Andrzej Massalski, Ulf Karsten

**Affiliations:** ^a^ M.H. Kholodny Institute of Botany, National Academy of Sciences of Ukraine, Tereschenkivska St. 2, KyivUA-01001, Ukraine; ^b^ University of Rostock, Institute of Biological Sciences, Applied Ecology and Phycology, Albert-Einstein-Strasse 3, D-18059Rostock, Germany; ^c^ University of Innsbruck, Institute of Botany, Functional Plant Biology, Sternwartestrasse 15, A-6020Innsbruck, Austria; ^d^ Jan Kochanowski University, Institute of Biology, Dept of Botany, Świetokrzyska St. 15, PL-25-406, Kielce, Poland

**Keywords:** *Interfilum*, *Klebsormidium*, molecular phylogeny, morphological characters, sporulation-like type of cell division, Streptophyta, ultrastructure

## Abstract

Representatives of the closely related genera, *Interfilum* and *Klebsormidium*, are characterized by unicells, dyads or packets in *Interfilum* and contrasting uniseriate filaments in *Klebsormidium*. According to the literature, these distinct thallus forms originate by different types of cell division, sporulation (cytogony) versus vegetative cell division (cytotomy), but investigations of their morphology and ultrastructure show a high degree of similarity. Cell walls of both genera are characterized by triangular spaces between cell walls of neighbouring cells and the parental wall or central space among the walls of a cell packet, exfoliations and projections of the parental wall and cap-like and H-like fragments of the cell wall. In both genera, each cell has its individual cell wall and it also has part of the common parental wall or its remnants. Therefore, vegetative cells of *Interfilum* and *Klebsormidium* probably divide by the same type of cell division (sporulation-like). Various strains representing different species of the two genera are characterized by differences in cell wall ultrastructure, particularly the level of preservation, rupture or gelatinization of the parental wall surrounding the daughter cells. The differing morphologies of representatives of various lineages result from features of the parental wall during cell separation and detachment. Cell division in three planes (usual in *Interfilum* and a rare event in *Klebsormidium*) takes place in spherical or short cylindrical cells, with the chloroplast positioned perpendicularly or obliquely to the filament (dyad) axis. The morphological differences are mainly a consequence of differing fates of the parental wall after cell division and detachment. The development of different morphologies within the two genera mostly depends on characters such as the shape of cells, texture of cell walls, mechanical interactions between cells and the influence of environmental conditions.

## Introduction

Over the past few decades, newly obtained molecular phylogenetic data have often conflicted with traditional systems based on algal morphology (Pröschold & Leliaert, 2007; Friedl & Rybalka, 2012). Genetic data are presumed to reflect the real phylogenetic relationships between organisms and to shed light on their origin during evolution. Hence, morphology has become less significant in taxonomic and floristic studies, but detailed morphological (including ultrastructural) investigations can often help to interpret molecular results such as unusual phylogenetic positions of some organisms. Polyphasic approaches based on comparisons of morphology, ultrastructure, molecular phylogenetics, ecophysiology and biochemistry have been used in recent taxonomic revisions of various groups of green and streptophycean algae (Pröschold *et al*., 2001, 2011; Karsten *et al*., 2005; Yamamoto *et al*., 2007; Eliáš *et al*., 2008; Mikhailyuk *et al*., 2008; Škaloud & Peksa, 2008; Darienko *et al*., 2010; Bock *et al*., 2011; Neustupa *et al*., 2011; Demchenko *et al*., 2012). Often, careful morphological and ultrastructural investigations undertaken several decades ago (Korshikov, 1938; Lokhorst, 1996; Lokhorst *et al*., 2000; Ettl, 1983; Tschermak-Woess, 1980*a*, *b*) have partly been confirmed by modern phylogenetic data (e.g. Pröschold *et al*., 2001; Eliáš *et al*., 2008; Sluiman *et al*., 2008; Škaloud & Peksa, 2010; Rindi *et al*., 2011; Demchenko *et al*., 2012). Combining data obtained by different methods (morphology, ultrastructure, molecular phylogeny) is important in algal taxonomy.

The Klebsormidiales (Streptophyta) contains the filamentous genera *Klebsormidium* P.S. Silva, Mattox & Blackwell, *Hormidiella* M.O.P. Iyengar & Khantamma and *Entransia* E.O. Hughes (Sluiman *et al*., 2008). *Klebsormidium* are typical filamentous algae, with cells dividing vegetatively (Floyd *et al*., 1972; Pickett-Heaps, 1972; Sluiman *et al*., 1989; Van den Hoek *et al*., 1995; Honda & Hashimoto, 2007; Katsaros *et al*., 2011). The Klebsormidiales also includes the genus *Interfilum* Chodat which is characterized by unicells and the formation of short filaments, dyads, packets or branched pleurococcoid filaments (Mikhailyuk *et al*., 2008). According to the original description, *Interfilum paradoxum* Chodat & Topali is an easily disintegrating filamentous alga, with cells surrounded by bipartite cell walls, dividing by vegetative cell division, forming chains of cells connected by ‘threads’ of unknown nature (Chodat & Topali, 1922). The cells divide by a process similar to sporulation; the remnants of parental walls form cap-like structures on the cells or thread-like structures between them, so cell walls appear bipartite (Mikhailyuk *et al*., 2008).

We conducted a detailed morphological and ultrastructural investigation of representatives of *Interfilum* and *Klebsormidium*, with emphasis on the protoplast and cell wall, the ‘behaviour’ of the cell wall during cell detachment, and the morphology of cell division, in an attempt to understand how different morphologies develop in two closely related genera.

## Materials and methods

### Strains and culture conditions

About 100 strains of *Interfilum* and *Klebsormidium* from the Sammlung von Algenkulturen, University of Göttingen, Germany (SAG: Friedl & Lorenz, 2012; www.epsag.uni-goettingen.de), the Culture Collection of Algae and Protozoa (CCAP, Gaсhon *et al*., 2007; www.ccap.uk), the collection from the project ‘Biota of South Africa’ (Büdel *et al*., 2009), the collection of *Klebsormidium* strains from Alpine soil crusts (Karsten *et al*., 2010; Holzinger *et al*., 2011; Kaplan *et al*., 2012; Karsten & Holzinger, 2012), some of our own isolates, and samples of field material were used for this study. Information about these strains is included in previous papers (Mikhailyuk *et al*., 2008; Rindi *et al*., 2011; Karsten *et al*., 2013). Information about the 30 strains presented here is summarized in Supplementary Table 1.

All cultures were grown on solid 1.5% agar or liquid modified Bold’s Basal Medium (3NBBM with vitamins; Starr & Zeikus, 1993), and kept at 20°C and 30–35 μmol photons m^−2^ s^−1^ under a light/dark cycle of 16:8 h L:D. Osram Daylight Lumilux Cool White lamps (L36W/840; Osram, Munich, Germany) were used as light sources.

### Light, fluorescent and laser scanning confocal microscopy, morphological characterization and vital staining

Young (2–3-week-old) and old (2–3-month-old) cultures of *Klebsormidium* and *Interfilum*, as well as field-collected material, were morphologically characterized using Olympus BX60 and Olympus IX70 light microscopes (Olympus Europe Holding, Hamburg, Germany) with Nomarski differential interference optics. The morphology of algae was documented with the attached Olympus ColorView III and II cameras (Soft Imaging System GmbH, Münster, Germany) using the imaging software Cell^D and analySIS (Soft Imaging System GmbH). An Olympus IX70 microscope equipped with a fluorescent lamp was used for investigation of mitochondria. A Leica TCS SP2 AOBS laser scanning confocal microscope (Leica Microsystems, Germany) was used for chloroplast morphology and mitochondria.

Mucilage was stained with an aqueous solution of methylene blue at different concentrations. For mitochondrial observations, cells were stained overnight with about 0.75 μg MitoTracker Green FM (Molecular Probes, Eugene, Oregon, USA) dissolved in 100 μl medium (3NBBM) with algal cells. The autofluorescence of chlorophyll was used to observe chloroplast structure (excitation at 488 nm, emission at 610–660 nm).

### Transmission electron microscopy

Samples from different phylogenetic clades according to Rindi *et al*. (2011) (clade A: SAG 338.1, SAG 2100, SAG 2101, SAG 2102; clade B/C: KUE1 and ASIB V100; clade D: PIT1 and SAG 5.96; clade E: SAG 2417, BOT3, SAG 2416, STR1; clade F: SAG 2415; clade G: 14613.5e) were fixed for transmission electron microscopy (TEM) using chemical fixation protocols according to Massalski *et al*. (1995) or Holzinger *et al*. (2009). For TEM, ultrathin sections were prepared, counterstained with uranyl acetate and Reynold’s lead citrate, and investigated in Zeiss LIBRA 120 or Tesla BS 500 transmission electron microscopes at 80 kV. Images were captured with a ProScan 2k SSCCD camera (Proscan Electronic Systems, Lagerlechfeld, Germany) and further processed using Adobe Photoshop software (Adobe Systems Inc., San José, California, USA).

### Phylogenetic data

The phylogenetic positions of the strains were obtained from earlier publications (Mikhailyuk *et al*., 2008; Rindi *et al*., 2011; Kaplan *et al*., 2012; Karsten & Holzinger, 2012; Karsten *et al*., 2013; Kitzing *et al*., 2014). Phylogenetic data for five *Klebsormidium* strains (HOH2, BRE, ASIB V100, PIT1, STR1) were obtained by T. Pröschold according to methods described in Karsten *et al*. (2013). Phylograms inferred from Maximum Likelihood analysis of the ITS rRNA in the Klebsormidiales published by Rindi *et al*. (2011) were used for designation of phylogenetic lineages within *Interfilum* and *Klebsormidium*.

## Results

### Light microscopy

**Figs 1–9.**
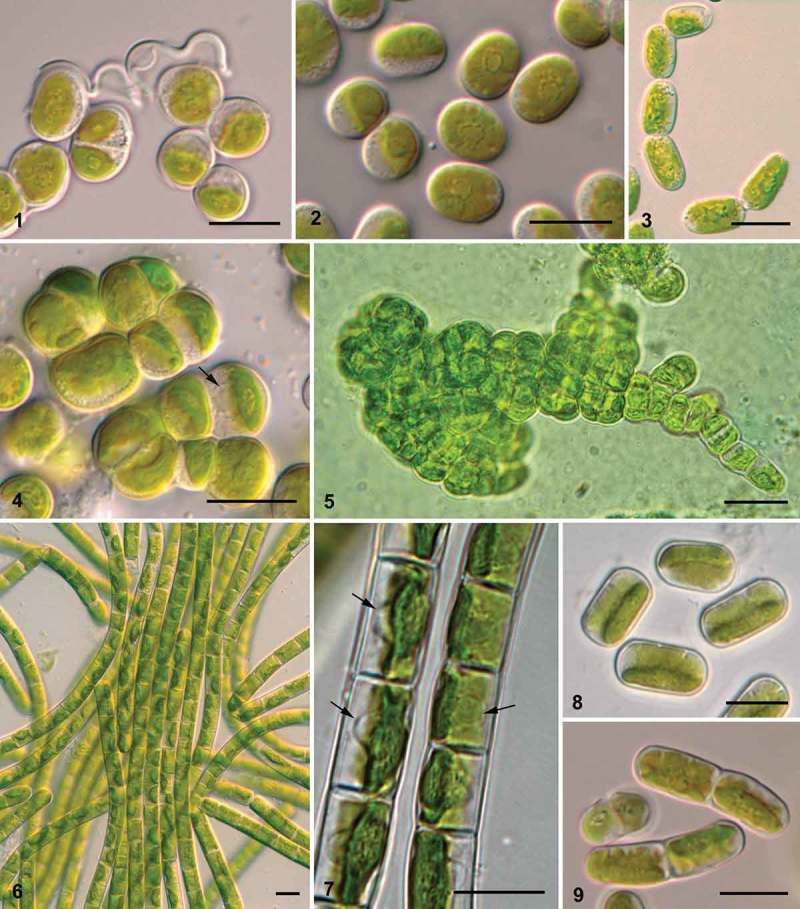
Diversity of morphotypes in *Interfilum* and *Klebsormidium*. **Fig. 1**. *Interfilum paradoxum* (SAG 338.1): unicells and dyads connected by ‘threads’. **Fig. 2**. *Interfilum* sp. (SAG 2101), unicells. **Fig. 3**. *Interfilum* sp. (SAG 36.88), short filaments. **Figs 4, 5**. *Interfilum massjukiae* (SAG 2102), packets and branched filaments. **Figs 6, 7**. Unbranched long filaments in *Klebsormidium* cf. *flaccidum* (Biof-4) (Fig. 6), and *Klebsormidium flaccidum* (ASIB V100) (Fig. 7). **Figs 8, 9**. Unicells and dyads in *Klebsormidium* cf. *dissectum* (TR 44) (Fig. 8) and *Klebsormidium* sp. (SAG 2108) (Fig. 9). Arrows indicate nuclei. Scale bars 10 μm.

Representatives of different lineages of *Interfilum* and *Klebsormidium* formed unicells, dyads, packets, cubic aggregates, and short and long uniseriate filaments, as well as biseriate parts and branched pleurococcoid thalli (Figs 1–9). The protoplast structure of the two genera was similar. Cells had one parietal chloroplast with smooth, undulating or variously dissected edges (Figs 1, 2, 4, 7–9) and a central pyrenoid surrounded by several or many starch grains (Figs 1, 2, 4). The nucleus was located opposite the pyrenoid (Fig. 7).

**Figs 10–21.**
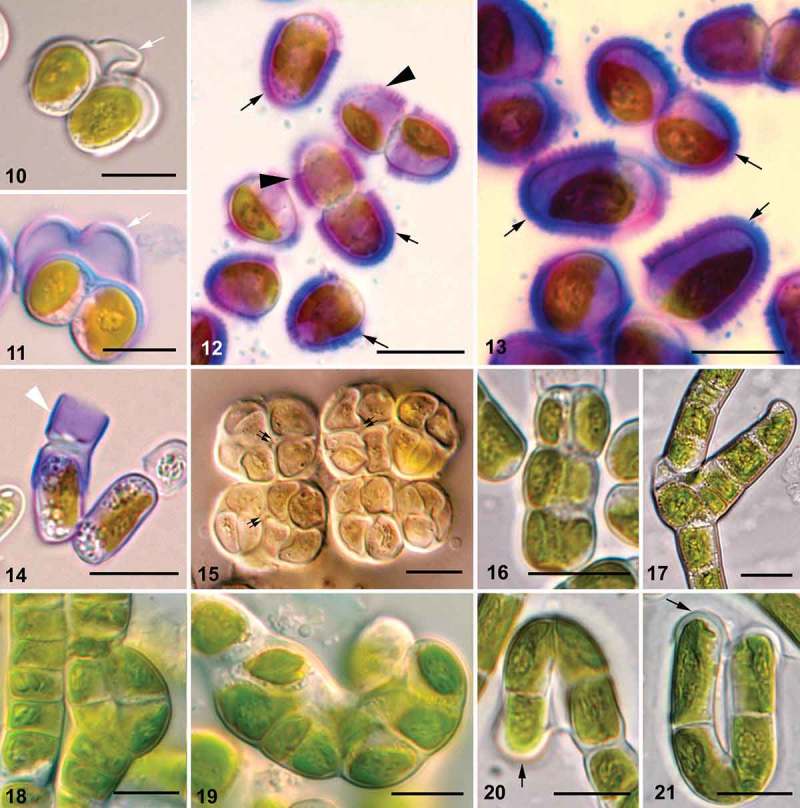
Morphology of *Interfilum* and *Klebsormidium* cell walls, ability of *Klebsormidium* cells to divide in several planes, and formation of branches. **Figs 10, 11**. Exfoliated parental walls forming ‘threads’ between cells (white arrows). **Figs 12, 13**. Cap-like (black arrows) and ring-like (black arrowheads) structures. **Fig. 14**. Stained H-like cell wall fragment in *Interfilum* (white arrowhead). **Fig. 15**. Spaces between cells in *Interfilum* packets (double black arrows). **Figs 16-19**. Biseriate parts of filaments, and packet- and branch-like structures in *Klebsormidium*. **Figs 20, 21**. Cap-like structures of *Klebsormidium* (black arrows). Material illustrated is as follows: Figs 10, 11, *Interfilum paradoxum* (SAG 338.1); Figs 12, 13, *Interfilum terricola* (SAG 2100); Fig. 14. *Interfilum* sp. (SAG 36.88); Fig. 15, *Interfilum* sp. (SAG 2147); Fig. 16, *Klebsormidium* cf. *subtile* (BRE); Fig. 17, *Klebsormidium nitens* (SAG 2417); Fig. 18, *Klebsormidium* sp. (TR 18); Fig. 19, *Klebsormidium* sp. (TR 24); Figs 20, 21, *Klebsormidium* cf. *subtile* (HOH2). Scale bars 10 μm.

Investigation of the *Interfilum* cell wall by light microscopy and mucilage staining showed the presence of cap- and ring-like structures as well as exfoliations of the parental wall, forming bridges between cells (Figs 10–13). H-like fragments of the cell wall were found occasionally (Fig. 14). Spaces between neighbouring cells were observed in packet-forming strains (Fig. 15). All these characters were related to the presence of individual walls in each cell and a parental wall.

*Klebsormidium* observed with light microscopy showed the rare presence of biseriate parts of filaments, packet- and branch-like structures in some strains, especially in old cultures (Figs 16–19). Cap-like structures (Figs 20, 21), H-like fragments of cell walls (Figs 22, 23, 25, 27, 28), and exfoliations of parental walls (Figs 24, 26), as well as triangular spaces between walls of neighbouring cells (Fig. 29), were frequently present. These structures were most obvious in field-collected material of *Klebsormidium*, which showed dense cell walls (Figs 30, 31).

**Figs 22–31.**
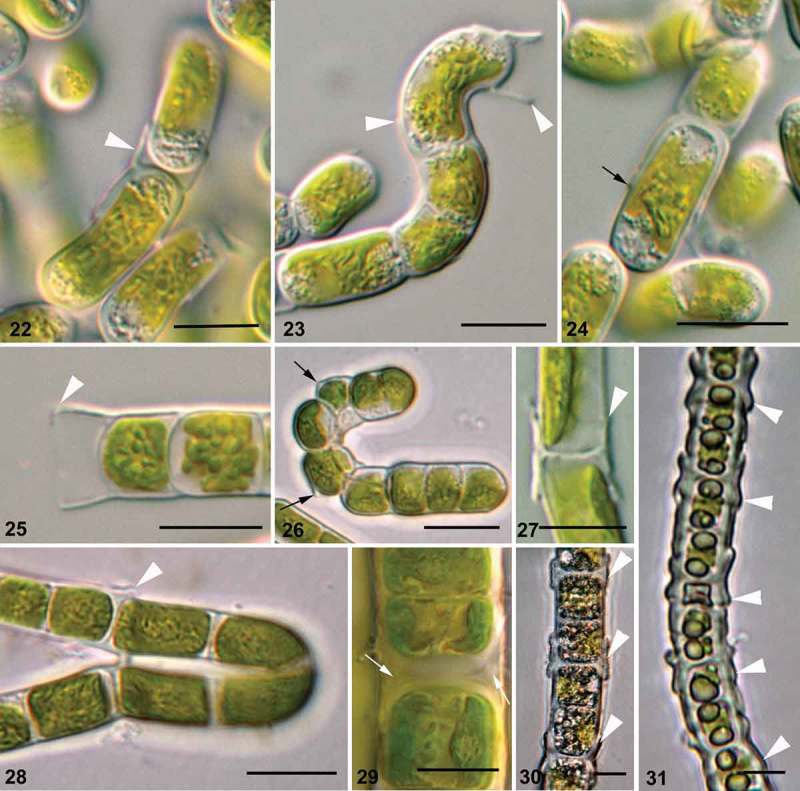
Structure of *Klebsormidium* cell wall on morphological level. **Figs 22, 23, 25, 27, 28, 30, 31**. H-like fragments of cell wall (white arrowheads). **Figs 24, 26**. Exfoliations of parental wall (black arrows). **Fig. 29**. Triangular spaces between daughter- and mother-cell walls (white arrows). Material illustrated is: Figs 22–24, *Klebsormidium* sp. (14621-6); Fig. 25, *Klebsormidium* cf. *flaccidum* (TR 42); Fig. 26, *Klebsormidium* cf. *subtile* (BRE); Fig. 27, *Klebsormidium* cf. *flaccidum* (SAG 12.91); Fig. 28, *Klebsormidium* cf. *subtile* (HOH2); Fig. 29, *Klebsormidium crenulatum* (SAG 37.86); Figs 30, 31, *K. crenulatum* (field-collected material). Scale bars 10 μm.

### Confocal laser scanning and fluorescence microscopy

Images obtained with confocal laser scanning and fluorescence microscopy showed that *Interfilum* and *Klebsormidium* strains contained the same type of variously lobed plate-shaped chloroplasts (Figs 32–35). Vital staining of cells of both genera revealed the presence of several polymorphic mitochondria, which were located around the nucleus and along the chloroplast lobes (Figs 36–42).

**Figs 32–42.**
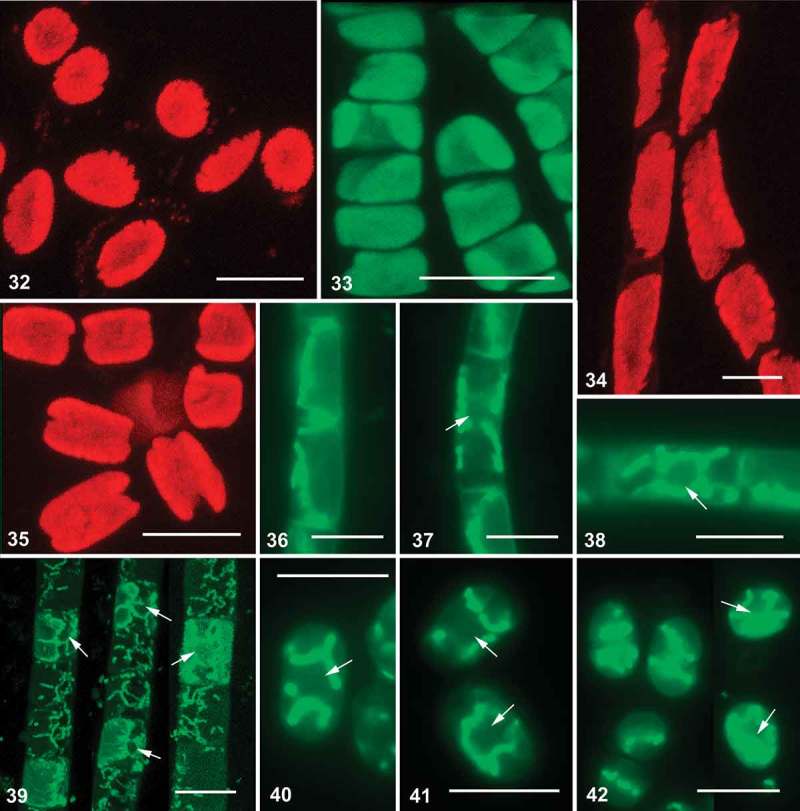
Confocal laser scanning and fluorescence micrographs of *Interfilum* and *Klebsormidium.* **Figs 32–35**. Autofluorescence of chloroplasts of different strains (confocal micrographs). **Figs 36–42**. Vital staining of mitochondria, arrows indicate position of nucleus (Figs 36–38, 40–42: fluorescence micrographs; Fig. 39: confocal micrograph). Material illustrated is: Figs 32, 42, *Interfilum terricola* (SAG 2100); Fig. 33, *Klebsormidium* sp. (14621.6); Fig. 34, *Klebsormidium subtile* (CCAP 335.17); Figs 35, 40, 41, *Klebsormidium* sp. (SAG 2107); Figs 36–38, *Klebsormidium fluitans* (CCAP 335.12); Fig. 39, *Klebsormidium* cf. *flaccidum* (TR 42). Scale bars 10 μm.

### Transmission electron microscopy

**Figs 43–50.**
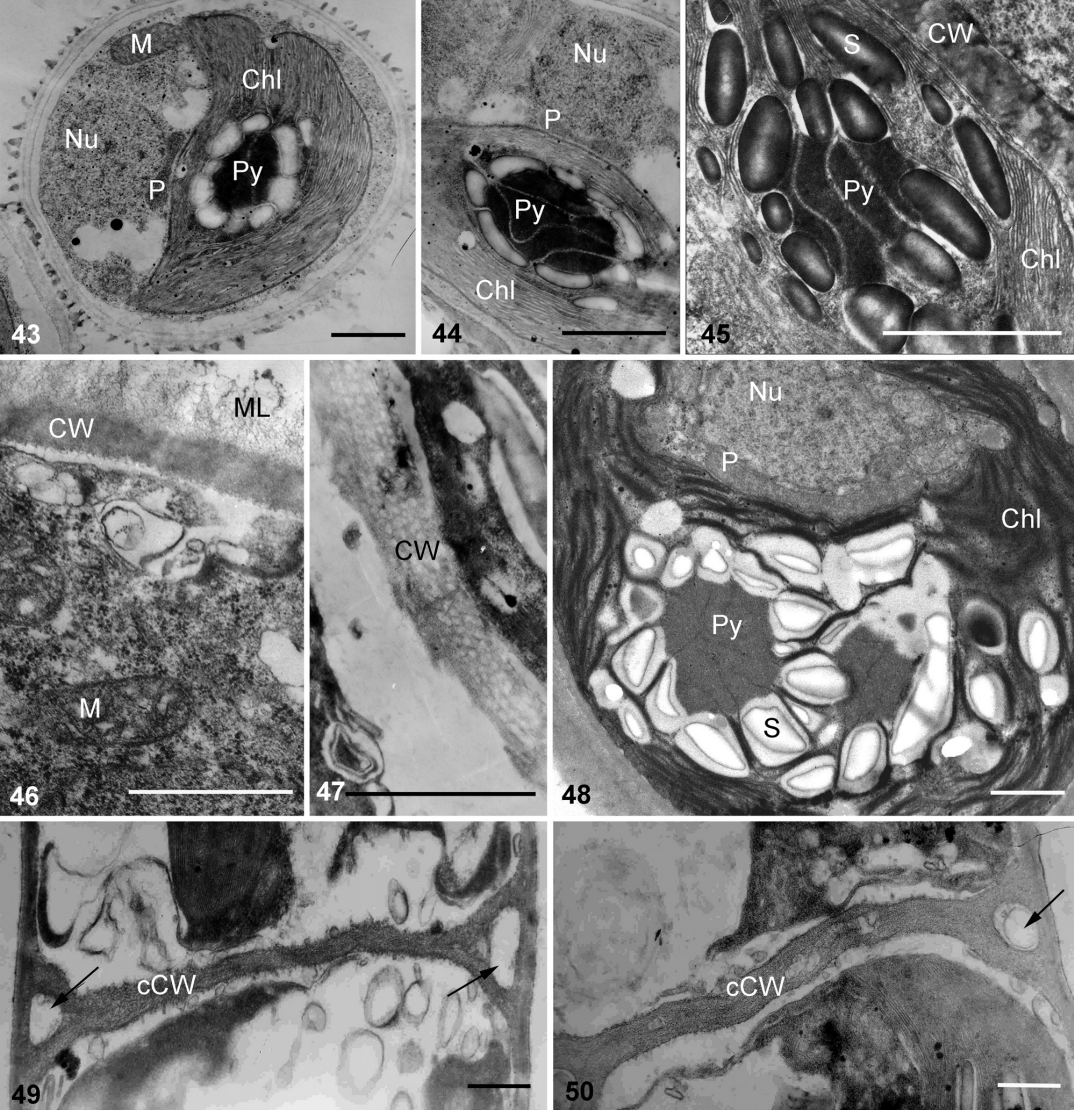
Transmission electron micrographs of vegetative cells and cell wall structure of *Interfilum* and *Klebsormidium*. **Figs 43–45**. Pyrenoid structure and position of peroxisome, nucleus and mitochondria in *Interfilum* cells. **Figs 46, 47**. Cell wall structure of *Interfilum* (homogeneous cell wall with mucilage (Fig. 46) and multilayered cell wall without mucilage (Fig. 47)). **Fig. 48**. Pyrenoid structure and position of peroxisome, nucleus and mitochondria in *Klebsormidium* cell. **Figs 49, 50**. Cross-cell wall structure of *Klebsormidium*; arrows indicate triangular spaces between the cell walls of daughter cells and the mother cell wall. Material illustrated is: Fig. 43, *Interfilum paradoxum* (SAG 338.1); Fig. 44, *Interfilum terricola* (SAG 2100); Figs 45, 46, *Interfilum* sp. (SAG 2101); Fig. 47, *Interfilum massjukiae* (SAG 2102); Fig. 48, *Klebsormidium crenulatum* (SAG 2415); Figs 49, 50, *Klebsormidium bilatum* (SAG 5.96). Abbreviations: Chl, chloroplast; CW, cell wall; cCW, cross-cell wall; ML, mucilage layer; P, peroxisome; Nu, nucleus; S, starch grain; M, mitochondrion; Py, pyrenoid. Scale bars 1 µm.

TEM investigations showed a similar pyrenoid ultrastructure in both genera (Figs 43–45, 48, 52, 53). Starch grains formed one or several layers around the pyrenoid body, arranged in parallel rows. Several to many parallel single thylakoid membranes penetrated the pyrenoid body, which determined the orientation of the starch grains. One peroxisome was located between the chloroplast and the nucleus (Figs 43, 44, 48). Mitochondrial profiles were located close to the chloroplast (Fig. 43).

**Figs 51–58.**
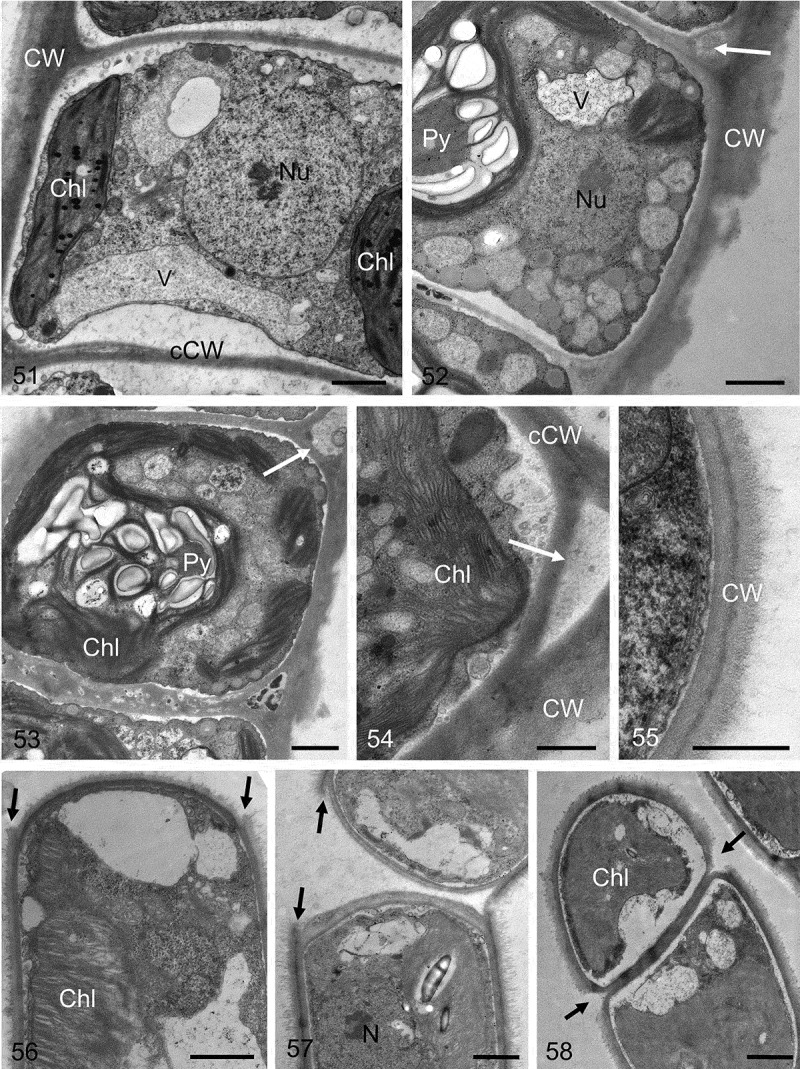
Transmission electron micrographs of different *Klebsormidium* strains. **Fig. 51**. Central nucleus and parietal chloroplast clearly visible. **Fig. 52**. Chloroplast contains pyrenoid, the outer cell wall exhibits clear layering with a corrugated outer surface, and the triangular space between the outer cell wall and the cross-wall is marked with a white arrow. **Fig. 53**. Pyrenoid with numerous starch grains, triangular space is marked with a white arrow. **Fig. 54**. Triangular space (white arrow) between the outer cell wall and the cross-wall. **Fig. 55**. Double-layered outer cell wall. **Fig. 56**. Terminal cell, showing the projections of the mother-cell wall (black arrows). **Fig. 57**. Two cells still not fully separated, showing the projections of the mother-cell wall (black arrows). **Fig. 58**. Initiating separation of two cells, the mother-cell wall is already separated (black arrows). Material illustrated is: Figs 51–54, *Klebsormidium crenulatum* (SAG 2415); Fig. 55, *Klebsormidium dissectum* (SAG 2416); Figs 56, 57, *Klebsormidium nitens* (SAG 2417); Fig. 58, *Klebsormidium* cf. *nitens* (STR1). Abbreviations: Chl, chloroplast; CW, cell wall; cCW, cell cross-wall; Nu, nucleus; Py, pyrenoid; V, vacuole. Scale bars Figs 51–53, 56–58, Figs 54–55: 1 µm; d, e: 500 nm.

Depending on the lineage, the *Interfilum* cell wall differed ultrastructurally. Unicellular species (*Interfilum terricola* (B. Petersen) Mikhailyuk, Sluiman, A. Massalski, Mudimu, Demchenko, T. Friedl & S.Y. Kondr. or *I. paradoxum*) had homogeneous cell walls with a fibrous mucilage layer (Fig. 46). Species forming cell packets (*I. massjukiae* Mikhailyuk, Sluiman, A. Massalski, Mudimu, Demchenko, T. Friedl & S.Y. Kondr.) were characterized by layered cell walls without mucilage (Fig. 47).

The cell walls of *Klebsormidium* strains showed triangular spaces between the cell walls of neighbouring cells (Figs 49, 50, 52, 54), as well as projections and exfoliations of the parental walls formed during cell detachment (Figs 56–58), and gelatinous parental walls similar to the cap-like structures of *Interfilum* (Figs 55, 58). All these characters indicate discontinuity of the *Klebsormidium* cell wall, and thus the presence of individual walls in each cell and parental wall. Cross-walls of *Klebsormidium* were bi- or multilayered, lacked plasmodesmata, and often showed different thicknesses within the same filament (Figs 51–53).

**Figs 59–66.**
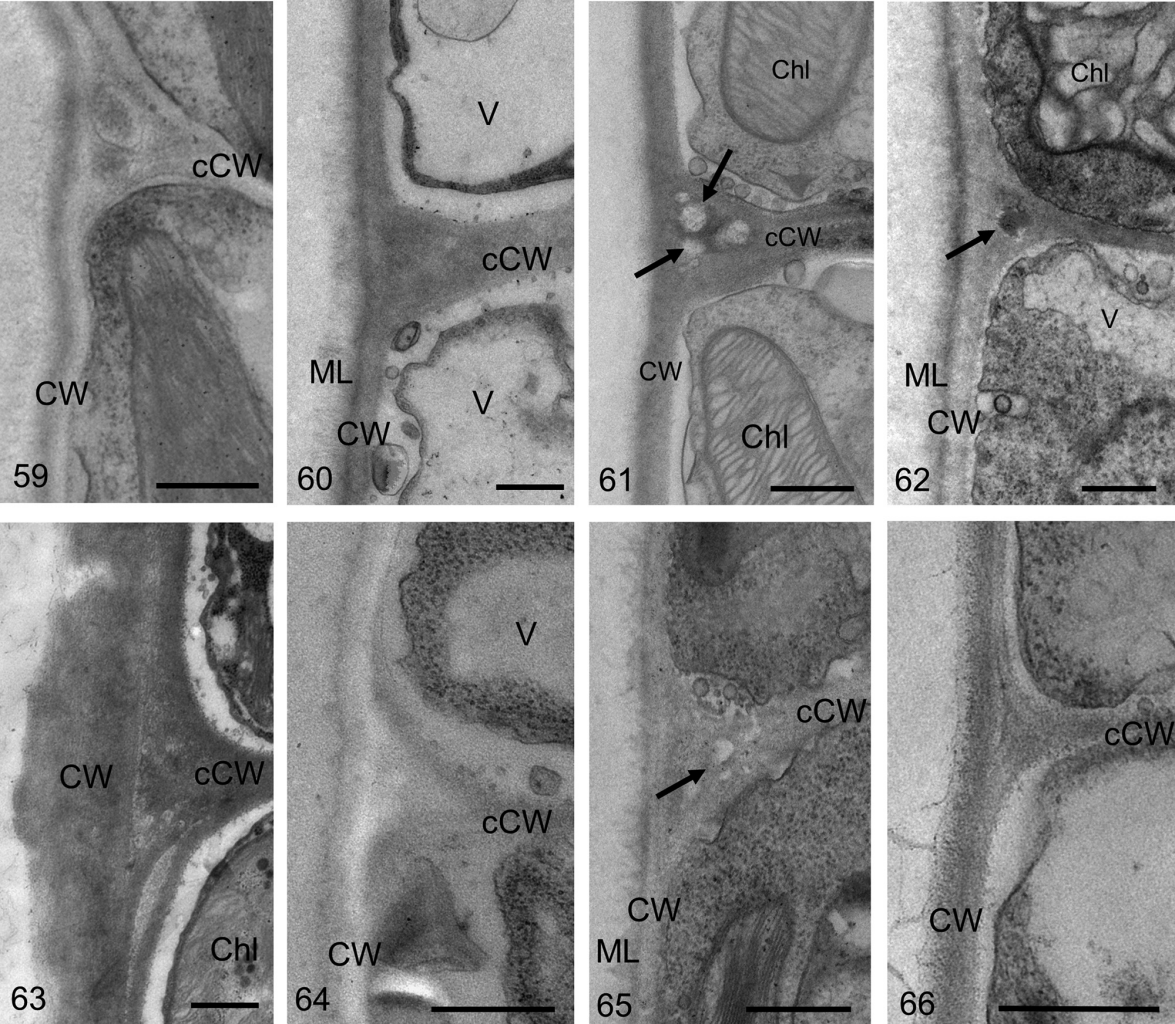
Transmission electron micrographs of different *Klebsormidium* strains; each image shows the left corners of two neighbour cells in a median section. The outer cell walls (CW) and cross-walls (cCW) separating the two cells are visible. **Fig. 59**. *Klebsormidium* cf. *flaccidum* (KUE1). **Fig. 60**. *Klebsormidium* cf. *flaccidum* (ASIB V100). **Fig. 61**. *Klebsormidium* sp. (14613.5e). **Fig. 62**. *Klebsormidium* cf. *bilatum* (PIT1). **Fig. 63**. *Klebsormidium crenulatum* (SAG 2415). **Fig. 64**. *Klebsormidium nitens* (SAG 2417). **Fig. 65**. *Klebsormidium* cf. *fluitans* (BOT3). **Fig. 66**. *Klebsormidium dissectum* (SAG 2416). Abbreviations: Chl, chloroplast; CW, cell wall; cCW, cell cross-wall; ML, mucilage layer; V, vacuole; arrows point to triangular spaces between the cell cross-walls on the edges. Scale bars 500 nm.

Ultrastructural analysis of cell walls in different lineages of *Klebsormidium* indicated overall similarity, but also some differences in detail (Figs 59–66). Samples from clades B/C KUE1 and ASIB V100 (Figs 59, 60) exhibited bilayered outer cell walls covered by a clearly distinguishable mucilage layer. The outer cell walls were ~ 0.16 µm thick in KUE1 and ~ 0.22 µm in ASIB V100, which together with the mucilage layer was ~ 0.4–0.5 µm in thickness (Figs 59, 60). The cell cross-walls were sometimes separated in KUE1, while in ASIB V100 they were rather thick and multilayered (Figs 59, 60). Representatives of clades D and G, PIT1 and 14613.5e (Figs 61, 62) showed bilayered outer cell walls with the outer layer less obvious in 14613.5e. The cell walls in PIT1 were ~ 0.17 µm thick, and in 14613.5e ~ 0.2 µm. In both strains, triangular spaces between the cross-walls and the outer cell wall were detectable (Figs 61, 62). The cross-walls were layered in 14613.5e, while they appeared rather smooth in PIT1. The thickest outer cell walls, in a *Klebsormidium* strain from clade F (*K. crenulatum* (Kützing) Ettl & Gärtner, SAG 2415), were up to ~0.6 µm thick, clearly layered and corrugated (Fig. 63). The cross-walls were also layered and clearly separated from the outer cell walls (Fig. 63). In many cases, triangular spaces between the outer cell walls and the cross-walls were visible (Figs 52–54). In contrast, *Klebsormidium* samples from clade E (*K. nitens* (Meneghini in Kützing) Lokhorst (SAG 2417), *K.* cf. *fluitans* (Gay) Lokhorst (BOT3) and *K. dissectum* (Gay) Ettl & Gärtner (SAG 2416), Figs 64–66) had rather thin cell walls, reaching 0.26 µm thickness in SAG 2417, 0.15 µm in BOT3 and 0.17 µm in SAG 2416. The cross-walls were thin and clearly separated from the outer cell wall in SAG 2416 (Fig. 66). Triangular spaces were observed only in BOT3 (Fig. 65). Layering of the cell walls and a mucilage layer were observed in all strains of clade E (Figs 64–66).

## Discussion

### Protoplast structure of *Interfilum* and *Klebsormidium*

According to our observations and to previously published data, protoplasts of *Interfilum* and *Klebsormidium* are characterized by common morphological and ultrastructural features: similar structures of the chloroplast and pyrenoid, position of the nucleus in a cytoplasmic bridge between the two terminal vacuoles, fibrous mucilage structure, shape and position of the single peroxisome, and mitochondria (Stewart *et al*., 1972; Silverberg, 1975; Lokhorst & Star, 1985; Morison & Sheath, 1985; Honda & Hashimoto, 2007; Mikhailyuk *et al*., 2008). The protoplast structure of some related streptophycean algae, e.g. members of *Entransia, Hormidiella, Chlorokybus* Geitler and *Coleochaete* Brébisson, is mostly similar as well (Rogers *et al*., 1980; Sluiman, 1985*a*; Lokhorst *et al*., 2000; Cook, 2004). Some conjugating green algae (Holzinger et al., 2009) and Anthocerotae mosses (Cook, 2004) have similar pyrenoid starch envelope structure. A large single peroxisome located between the chloroplast and the nucleus is also characteristic of *Mesostigma* Lauterborn, *Chaetosphaeridium* Klebahn, *Chlorokybus, Coleochaete* and *Hormidiella* (Rogers *et al*., 1980; Sluiman, 1985*a*; Melkonian, 1989; Van den Hoek *et al*., 1995; Lokhorst *et al*., 2000). Multiple peroxisomes of other shapes, closely adjacent to the chloroplast, are found in *Nitella* C. Agardh (Silverberg & Sawa, 1973), *Micrasterias* C. Agardh ex Ralfs (Tourte, 1972), photosynthetic cells of *Polytrichum* (Proctor *et al*., 2007) and vascular plants (Raven *et al*., 2005). The peroxisome, nucleus, chloroplast and mitochondria form a specific structural complex, which has been suggested to be a diagnostic feature for streptophycean green algae (Massalski, 2002; Massalski & Kostikov, 2005). The close arrangement of these organelles in the cell is considered to be an evolutionarily progressive character, because it guarantees rapid metabolic exchange processes during photorespiration (Raven *et al*., 2005). In addition, the enzyme composition of peroxisomes of streptophycean algae and embryophytes is similar, and differs from other algae (Gross, 1993). The structural complex plays an important role in cell division of streptophycean algae (see below). It is likely that the presence of this structural complex reflects the evolutionary success of this algal group (worldwide distribution in terrestrial ecosystems; Rindi *et al*., 2009), and hence is partially retained in embryophytes (Raven *et al*., 2005; Proctor *et al*., 2007).

Mitochondria are probably components of the structural complex as well. There are abundant TEM data on mitochondrial profiles in sections of *Klebsormidium* cells (Stewart *et al*., 1972; Silverberg, 1975; Lokhorst & Star, 1985; Morison & Sheath, 1985; Honda & Hashimoto, 2007), but little information about their spatial organization is available. Our data indicate that the location of mitochondria is always similar and strictly ordered. This spatial distribution may support the proposal that streptophycean algae contain a structural complex consisting of the peroxisome, nucleus, mitochondria and chloroplast. Small differences in mitochondrial location among different strains mostly depend on the cell shape and details of chloroplast morphology.

### Structure of cell walls and possible origin of cell wall remnants

Originally *Interfilum* was described as a genus with bipartite cell walls, closely related to the genus *Radiofilum* Schmidle (Chodat & Topali, 1922). Detailed morphological investigations by Fritsch & John (1942) showed that each *Interfilum* cell has its own integral cell wall, which is formed inside the parental wall during cell division. The parental wall ruptures in the middle during cell growth and detachment, resulting in cap-like structures closely associated with daughter cell walls. The origin of cap-, ring- and thread-like structures from parental wall remnants was shown in the present study and in a previous publication (Mikhailyuk *et al*., 2008).

Some cell wall structures observed here in *Klebsormidium* (H-like and cap-like structures, exfoliations and projections of the parental wall, triangular spaces between the parental wall and the cell walls of neighbouring cells) indicate that the cell wall is heterogeneous and includes daughter walls with closely adhering remnants of the parental wall. The H-like fragments represent structures homologous to the cap-like remnants characteristic of *Interfilum*: two cap-like structures connected by their tops. H-like fragments are observed in some filamentous algae with bipartite (*Microspora* Thur., *Tribonema* Derbès et Solier) or separated (consisted of daughter and parental walls) cell walls (*Binuclearia* Wittrock, *Cylindrocapsa* Reinsch) (Sluiman *et al*., 1989; Massjuk, 1993; Sluiman, 1985*b*; Van den Hoek *et al*., 1995), and probably in *Klebsormidium* as well. Cap-like structures characteristic of *Interfilum* were recently reported in some strains of *Klebsormidium* by Škaloud & Rindi (2013).

H-like fragments of cell walls are well known in *Klebsormidium* (Starmach, 1972; Moshkova, 1979; Ettl & Gärtner, 1995; Van den Hoek *et al*., 1995; Lokhorst, 1996), but their origin has not been explained satisfactorily. Lokhorst (1996) indicated that H-fragments are remnants of the parental wall, but did not explain how they are formed in an alga with vegetative cell division (only cross-walls are formed during this kind of cell division, as described below, without formation of separate daughter cell walls). Some proposed explanations indicate occasional formation of akinetes or hypnospores in *Klebsormidium* (Moshkova, 1979; Morison & Sheath, 1985). In this case, the cell protoplast forms its own cell wall, and the resting cell remains enclosed inside the parental wall. However, according to other authors (Klebs, 1896; Lokhorst, 1996), specialized resting cells are not formed in *Klebsormidium* because cells neighbouring H-fragments are usually not distinguishable (judging by the presence and location of cellular organelles) from other vegetative cells of the filament. Jane & Woodhead (1941) reported that field-collected filaments of *Ulothrix* Kützing and *Klebsormidium* contain many H-fragments of cell walls. Detailed microscopic investigation of the filaments showed that their cell walls are discontinuous, with distinct inner and outer layers. Microchemical tests revealed that these layers differ in chemical composition: whilst the inner layer consists of cellulose, the outer layer represents an intermediate stage in the degradation of cellulose to mucilage. Consequently, it is possible to interpret these layers as the daughter cell wall and gradually degrading remnants of the parental wall.

### Formation of packets, biseriate parts of filaments and branches in *Interfilum* and *Klebsormidium*

Some *Interfilum* strains are able to form packets, cubic cell aggregations and branched pleurococcoid thalli. Cells divide in several planes and remain enclosed within widened parental walls; the central space between cells of a packet observed in this study indicates that each daughter cell has its own cell wall.

The formation of packets, biseriate parts of a filament and branching have rarely been reported in *Klebsormidium* (Ettl & Gärtner, 1995; Lokhorst, 1996). The latter author interpreted these structures mostly as the formation and further germination of aplanospores, which formed one per cell and often remained inside the sporangial wall. Germination of the aplanospores led to the formation of branches or cell complexes. Lokhorst (1996) expressed some doubts concerning this interpretation, but proposed that this ability of *Klebsormidium* might be the first step in the development of definite side-branching.

Some examples of this branching are actually caused by germination of cells originating from zoospores (hemizoospores/aplanospores) inside a sporangial wall. Germination of these cells inside the sporangium leads to the formation of young filaments growing from a parental filament. This type of pseudobranching is clearly visible in some published figures (Lokhorst, 1996, figs 36, 75, 97, 186; Škaloud, 2006, fig. 8). Pseudobranches originating from reproductive cells are evident in micrographs of *Entransia fimbriata* E.O. Hughes (Cook, 2004, figs. 5e, f), which is closely related to *Klebsormidium*. This observation provides clear evidence that small branches can originate from reproductive cells, as the tip (characteristic of germinating zoospores of *E. fimbriata* and absent in *Klebsormidium*) is clearly visible on the branches.

However, the formation of biseriate parts, packet-like structures and some other kinds of branches in *Klebsormidium* has another origin, i.e. the division of vegetative cells occurs in several planes. This behaviour was observed in the present study and has been reported in the literature (Ettl & Gärtner, 1995, fig. 203d; Lokhorst, 1996, figs 188, 203, 207, 224). It seems that the cells divide in the same way as packet-forming strains of *Interfilum*.

**Fig. 67.**
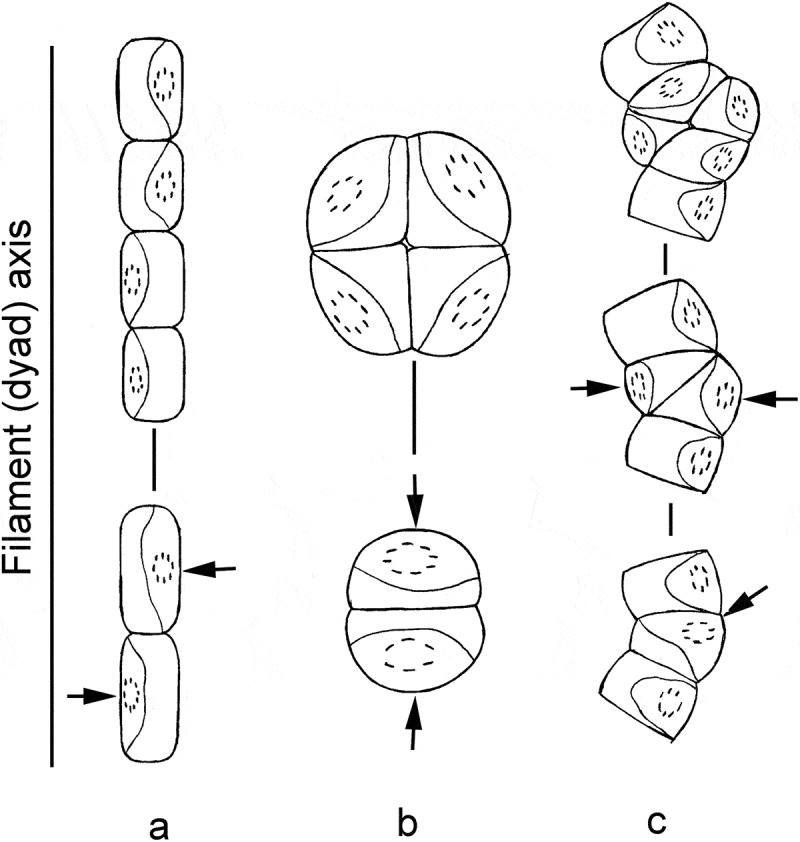
Scheme of cell division in *Klebsormidium* and *Interfilum*. a: division of cylindrical *Klebsormidium* cells, with formation of normal filament; b: division of almost spherical or wide-ellipsoid *Interfilum* cells, with formation of a packet; c: division of deformed *Klebsormidium* cell, with formation of biseriate part and packet-like structure. Arrows indicate the site of septa formation and plane of cell division.

It is known that the division of chloroplasts, together with the closely adhered peroxisome, occurs before division in vegetative cells of *Klebsormidium* and some other streptophycean algae (*Coleochaete*) (Floyd *et al*., 1972; Pickett-Heaps *et al*., 1972; Lokhorst & Star, 1985; Van den Hoek *et al*., 1995; Honda & Hashimoto, 2007). Furthermore, the septum dividing two protoplasts forms at the same position in the cell where cleavage of these organelles had occurred: the central part of the chloroplast and the centre of the structural complex, where the pyrenoid is located. Therefore, the site of septum formation is strongly correlated with the chloroplast position. Chloroplasts of *Klebsormidium* are usually located laterally, near longitudinal walls of cylindrical cells and parallel to the filament axis. Chloroplasts are maximally exposed to light in this position. Therefore, a septum dividing two daughter cells is formed in the plane perpendicular to the filament axis (Fig. 67a), resulting in a chain of cells. Cells of packet-forming members of *Interfilum* range from nearly spherical to broadly ellipsoid. Chloroplasts in the cell dyad are usually located perpendicular to the dyad axis. This part of the cell wall is the longest, and the chloroplasts are maximally exposed to light. The septum is formed similarly to that in *Klebsormidium*, but parallel to the dyad axis and perpendicular to the previous cross-wall (Fig. 67b). A four-celled packet is formed as a result.

Therefore, the location of the chloroplast (or structural complex) and, partly, the shape of the cell determine the plane of cell division in *Interfilum* and *Klebsormidium*. Interestingly, some fundamental rules concerning cell division in embryophytes indicate that the plane of cell division is correlated with the shape of a cell: new cell walls normally form perpendicularly to the axis of growth of a cell (Dupuy *et al*., 2010). Therefore, the cross-dividing walls are normally formed in long cells, and longitudinal walls in short cells. Division in several planes does not occur in *Interfilum* strains with long cells. Occasional formation of biseriate parts was observed in *Klebsormidium* with short cells, i.e. *K. crenulatum* (Lokhorst, 1996) and *K. montanum* (Hansgirg) S. Watanabe (Ettl & Gärtner, 1995). We usually observed these structures in old cultures of *Klebsormidium*, when the cells became shorter. Sometimes the formation of biseriate parts of filaments is related to the deformation of cells, causing a curvature of the filament. The chloroplast has too little space for lateral dislocation in short or deformed cells, and hence sometimes turns from the short lateral wall to the longer cross-wall, or locates obliquely. The septum forms according to the usual rule, but perpendicularly or obliquely to the filament axis (Fig. 67c), in this case forming biseriate parts, packet-like structures or branches. Cell division in several planes in both *Interfilum* and *Klebsormidium* shows clearly that many of the properties of dividing plant cells are influenced physically or mechanically (Dupuy *et al*., 2010).

### Type of cell division in *Interfilum* and *Klebsormidium*

Various modes of cell division of algae have been described. However, at present two main types of division can be distinguished among green algae with rigid cell walls: sporulation (cytogony, eleutheroschisis) and vegetative cell division (cytotomy, desmoschisis) (Ettl, 1988*a*, *b*; Sluiman *et al*., 1989). The mechanism of cytokinesis of the two types differs substantially and leads to the formation of different division products: specialized reproductive cells (spores or gametes) or young vegetative cells.

The fundamental morphological characters distinguishing sporulation and vegetative cell division are distinctive features in the formation of daughter cell walls. The cross-wall is always formed *de novo*, but longitudinal cell walls of parental cells are preserved and become part of the daughter cell wall during vegetative cell division (Ettl, 1988*a*, *b*; Massjuk, 1993; Van den Hoek *et al*., 1995). Therefore, the cell wall of a thallus is integral, since each cell is part of the multicellular organism. Pores with plasmodesmata are often characteristic of cross-walls of multicellular algae, in which separate cells are united in an integrated organism (Van den Hoek *et al*., 1995). The main characteristic of sporulation is the formation of cell walls by each cell independently within the parental (sporangial) wall (Ettl, 1988*a*, *b*; Sluiman *et al*., 1989). The protoplast of the parental cell divides into several parts, each forming its own cell wall during sporulation. The wall of the parental cell is transformed in different ways: it may degrade, releasing the cells to form unicellular organisms, or remain intact, holding the cells in colonies and forming extracellular structures. The cell walls are discontinuous and the cells are not connected by plasmodesmata, although they are united in multicellular complexes that represent colonies of unicellular organisms (Sluiman *et al*., 1989).

The type of cell division of many algae is easy to attribute to sporulation because it leads to the formation of a typical unicellular state. Cell division of packet-forming (sarcinoid morphotype) and filamentous algae traditionally was regarded as vegetative cell division (Fritsch, 1935; Smith, 1955). However, the morphological classification created by Ettl (1988*a*, *b*) and supported with some ultrastructural characters by Sluiman *et al*. (1989) determined that cell division of many filamentous and sarcinoid algae is actually a kind of sporulation. A complex of ultrastructural and morphological features (mostly accompanied by the presence of partially reduced flagellar structures in daughter cells, specific origin of the septum plasma membrane and a post-cytokinetic circumferential deposition pattern of extracellular material) were used as diagnostic characters indicating cell division via sporulation. The ultrastructural characters of the above-mentioned spores are completely reduced in some algae. The last character (circumferential deposition of the new cell wall) is the most important, as it indicates the formation of a daughter cell wall within the parental wall and attributes cell division to the sporulation type. Cell division of some packet-forming (*Chlorosarcinopsis* Herndon, *Tetracystis* R.M. Brown & H.C. Bold, *Trebouxia* Puymaly) and filamentous algae (*Geminella* Turpin, *Binuclearia, Cylindrocapsa, Nannochloris* Naumann, *Marvania* F. Hindák, *Microspora, Stichococcus* Nägeli, *Oedogonium* Link ex Hirn) was determined to be sporulation on the basis of these characters (Sluiman, 1985*b*; Sluiman & Reymond, 1987; Sluiman & Lokhorst, 1988; Sluiman *et al*., 1989; Yamamoto *et al*., 2007). The next conclusion derived from this classification was a fundamentally different concept of thallus organization in these algae: colonies of unicellular organisms (packets or pseudofilaments) and their vegetative cells are spores, according to their origin (Ettl, 1988*a*, *b*; Sluiman *et al*., 1989).

Although this classification system of cell division is generally accepted (Van den Hoek *et al*., 1995), there are also contradictory arguments (Massjuk, 1997, 2000; Massjuk & Demchenko, 2001), i.e. that the products of the cell division are not spores or gametes, but rather vegetative cells. A wider concept of vegetative cell division was proposed later: ‘During vegetative cell division the parental cell wall does not rupture or gelatinize, but is used for organization of daughter cell walls, which leads to the formation of vegetative (somatic, not specialized reproductive) cells and growth of thallus’ (Massjuk, 2000). This concept has some debatable points as well. Although parental cell walls in pseudofilamentous algae usually represent support for the daughter cell and are involved in the formation of a thallus, they degrade via rupture or gelatinization and transform into remnants (Sluiman, 1985*b*; Sluiman & Reymond, 1987; Yamamoto *et al*., 2007).

The cell division of packet-like and pseudofilamentous algae is a transitional type between sporulation and vegetative cell division. The mechanism of this cell division must be attributed to sporulation: the daughter cell wall forms within the parental wall, and the parental wall represents different stages of degradation. The products of cell division (somatic cells, not spores or gametes) reflect vegetative cell division. The parental cell wall is partially retained after this cell division and participates in the formation of the thallus. In our opinion, the best definition of cell division within packet-like and pseudofilamentous algae is ‘desmoschisis’ *sensu* Groover & Bold (1969), as previously proposed by Massjuk (2000). However, this term is not generally accepted in modern phycological literature, and is considered a synonym of classical vegetative cell division (Sluiman *et al*., 1989).

Although cell division in *Interfilum* initially was determined to be vegetative cell division (Chodat & Topali, 1922), Fritsch & John (1942) reported a sporulation-like type of formation of daughter cell walls. The latter authors even considered *I. paradoxum* to be a colonial member of the Chlorococcales. The characters of *Interfilum* cell walls obtained in the present study are arguments for the formation of daughter cell walls within parental walls. Therefore, a sporulation-like type of cell division is characteristic for *Interfilum.*

Vegetative cells of *Klebsormidium* divide by vegetative cell division according to the traditional point of view (Floyd *et al*., 1972; Pickett-Heaps, 1972; Lokhorst & Star, 1985; Sluiman *et al*., 1989; Van den Hoek *et al*., 1995; Lokhorst, 1996). The cell cross-walls form by cleavage furrow and lack plasmodesmata (Lokhorst & Star, 1985; Van den Hoek *et al*., 1995; Lokhorst, 1996). However, different kinds of remnants of parental cell walls and triangular spaces between parental walls and cell walls of neighbouring cells are structures characteristic of the sporulation-like type of cell division, and are homologous to structures observed in *Interfilum*. Therefore, the type of cell division within the two genera must be the same. In addition, the presence of cross-walls with an H-like appearance (*Microspora*-type) is an argument for cell division by the sporulation-like type (Sluiman *et al*., 1989).

### Transformation of parental wall and probable structure of thalli in *Interfilum* and *Klebsormidium*

**Fig. 68.**
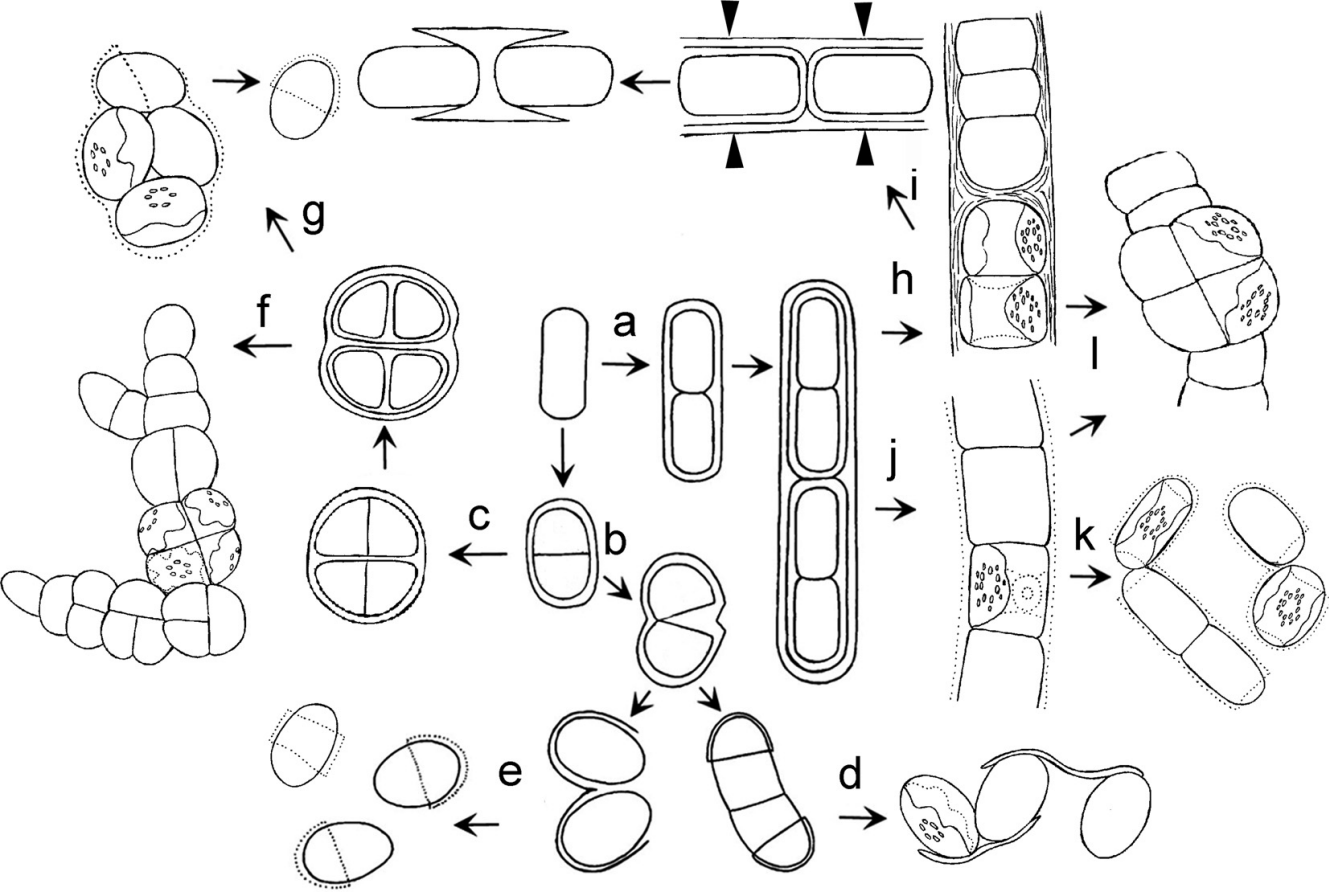
Scheme showing different routes of transformation of the *Interfilum* and *Klebsormidium* parental-cell wall, leading to formation of different morphotypes. a: preservation of parental wall, cylindrical cells divided mostly in one plane; b: gelatinization or rupture of parental wall; c: preservation of parental wall, almost-spherical cells divided in several planes; d: formation of unicells connected by ‘threads’; e: formation of unicells with cap- and ring-like structures; f: formation of packets and branched thallus; g: gelatinization of parental wall and formation of unicells with cap-like structures; h: preservation of parental wall, formation of strong filaments; i: formation of H-like fragments; j: partial gelatinization of parental wall; k: formation of short filaments, dyads and unicells; l: occasional division in several planes, formation of packet-like structures and biseriate parts. Arrowheads indicate sites of rupture of parental wall.

Gelatinization of parental walls leads to the formation of a mucilage envelope and disintegration of the thallus to the unicellular state. The cell walls of these *Interfilum* strains are usually thin and homogeneous because they represent a daughter cell wall. Preservation of parental walls around daughter cells caused the formation of densely layered walls, retaining cellular packets. The cell walls of these *Interfilum* strains are thick and layered, because they consist of a daughter cell wall and several generations of parental walls and their remnants. Partial rupture of the parental walls leads to the formation of cap-, ring- and thread-like structures. The left part of the scheme (Fig. 68b–g) shows that different routes of transformation of *Interfilum* parental walls lead to the formation of a specific thallus structure.

Vegetative cells of *Klebsormidium* usually divide in one plane, with subsequent formation of filamentous thalli. The scheme of a filament typical for other algae with the sporulation-like type of cell division (*Geminella, Binuclearia, Cylindrocapsa*; Massjuk, 1993; Van den Hoek *et al*., 1995) was chosen as a model of *Klebsormidium* filaments. The parental walls of *Klebsormidium* are partially preserved around two daughter cells during cell division (Fig. 68a). Further division of these cells proceeds in the same way, i.e. a chain of cells surrounded by many generations of parental walls is formed (Fig. 68a, h, j). Generations of parental walls are transformed in different ways (ruptured or gelatinized) because of the pressure of growing cells and remnants over time. This model corresponds to some characters of a *Klebsormidium* thallus and explains the formation of H- and cap-fragments of the cell wall. The different widths of cross-walls within the same filament of *Klebsormidium* is a further confirmation of this scheme; i.e. the cell walls are thin and bilayered between freshly divided cells, and thick and multilayered between groups of dividing cells.

The morphological diversity within *Klebsormidium* reflects different routes of further transformation of parental walls. The preservation of these parental walls around daughter cells leads to the formation of dense filaments, often with H-fragments (Fig. 68h). The mechanism for the formation of an H-fragment is as follows: the cell cross-wall is pressed from opposite sides by two growing neighbouring cells, and the layers of parental cell walls are compressed. Longitudinal cell walls, in contrast, are stretched and become thinner. The filaments are ruptured first in the middle of the longitudinal walls, and the cross-wall is preserved and forms an H-fragment (Fig. 68i). H-fragments are usually formed within the thickest cross-walls, i.e. between groups of dividing cells.

A second route of transformation of the *Klebsormidium* parental wall leads to gelatinization and formation of a delicate mucilage envelope around the filament (Fig. 68j). The filament can easily disintegrate into short filaments, and the unicells are sometimes morphologically similar to unicellular strains of *Interfilum*. These *Klebsormidium* strains sometimes form H-fragments, but this phenomenon is observed rarely because their parental walls are thin and delicate, and gelatinization of cross-walls leads to easy separation of cells. Biseriate and packet-like parts of filaments occur occasionally in *Klebsormidium* because of an atypical position of the chloroplast in the cell (Fig. 68l).

**Fig. 69.**
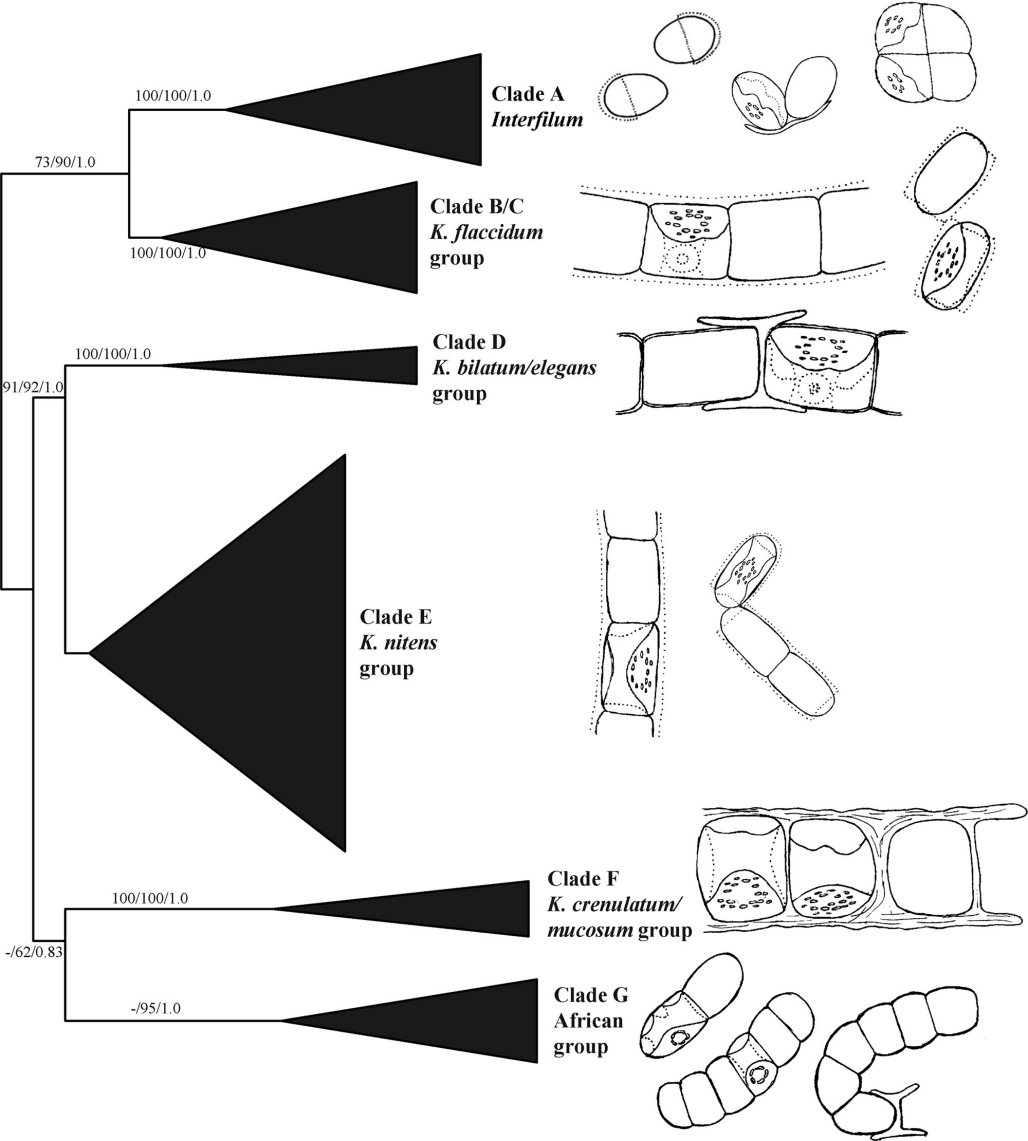
Schematic phylogenetic tree inferred from Maximum Likelihood analysis of the ITS rRNA in the Klebsormidiales, with bootstrap support (BP) and Bayesian posterior probabilities (PP) indicated at the nodes; from left to right the support values correspond to Neighbour Joining BP, Maximum Likelihood BP and Bayesian PP (Rindi *et al*., 2011, fig. 1). Right part of the figure shows schematic pictures of representatives corresponding to each clade.

Investigations of the ultrastructure of *Klebsormidium* cell walls of different phylogenetic lineages showed a high structural similarity, with some differences in details. These details are the width and degree of lamination of the cell walls, as well as the presence or absence of mucilage. Therefore, the diversity of structures and textures of *Klebsormidium* cell walls probably represents two main types: (1) dense filaments without mucilage (mostly characteristic of representatives of xerophytic lineages, i.e. clades D, F and G according to Rindi *et al*., 2011; Fig. 69), and (2) filaments with gelatinized cell walls that easily transform into short filaments or into the unicellular state (characteristic of mesophytic and hydrophytic lineages, i.e. clades E and partially B and C; Fig. 69).

In summary, the data presented here indicate a high similarity in the morphology and ultrastructure of vegetative cells and cell walls of *Interfilum* and *Klebsormidium*, and this similarity is in concordance with results of a recent phylogenetic analysis (Rindi *et al*., 2011). The different morphology of these genera is mostly a consequence of the different ‘behaviour’ of parental walls after cell division and detachment, as well as of the shape of the vegetative cells. Therefore, the presence of different morphotypes within the two genera depends on shape of cells, mechanical interactions between cells and the influence of environmental conditions.

### Structure of cell wall and possible type of cell division in other green and streptophycean algae

Cell walls of two other genera of Klebsormidiales, *Entransia* and *Hormidiella* Iyengar & Kanthamma, exhibit similar characters to those found in *Interfilum* and *Klebsormidium*. Triangular spaces between daughter and parental walls are visible in TEM micrographs of vegetative cells of *E. fimbriata* (Cook, 2004, figs. 6c, 7g). H-fragments of the cell wall are characteristic for this species (Cook, 2004). H-fragments are unknown for *H. attenuata* Lokhorst (Lokhorst *et al*., 2000), but we observed these structures in strain CCAP 329/1 cultivated on agar medium (data not shown). Consequently, cell division in *Entransia* and *Hormidiella* seems to be similar to that in *Interfilum* and *Klebsormidium*.

Triangular spaces between cells and the clear parental wall surrounding cell packet are visible in TEM micrographs of another streptophycean alga, *Chlorokybus atmophyticus* Geitler (Lokhorst *et al*., 1988, fig. 5). This species was mentioned as the sole representative among species formerly assigned to Charophyceae that shows a type of cell division intermediate between vegetative cell division and sporulation (Sluiman *et al*., 1989).

Investigation of species of *Coleochaete* cultivated under conditions simulating the terrestrial habitat (on solid medium and on sand grains) showed the formation of packet-like cell aggregates instead of the typical radial thalli (Graham *et al*., 2012). Cell packets are formed because of preservation of the parental walls (Graham *et al*., 2012, fig. 3c). It seems that vegetative cells of *Coleochaete* divide by the sporulation-like type because of the presence of the parental wall. However, the ultrastructural characters of cytokinesis in *Coleochaete* are completely different (formation of the cell plate in a phragmoplast) from those seen in the members of Klebsormidiales or Chlorokybales (cleavage furrow) (Lokhorst *et al*., 1988; Van den Hoek *et al*., 1995). These data show that a transition between two fundamentally different thallus structures or morphotypes (radial plate and sarcinoid packet) occurs in nature more often than was previously thought, and depends on environmental conditions. In general, the formation of cell packets and cubic aggregations is typical for many terrestrial algae, and is related to a reduction of the cell surface area subject to evaporation (Nienow, 1996; Karsten *et al*., 2010). The example of *Coleochaete* shows that the formation of a packet-like morphotype might be an adaptation to terrestrial conditions.

The tendency for cells to easily transition to divide in three planes and form packet-like cell aggregates is probably typical for the sporulation-like type of cell division. This phenomenon was observed within the clade ‘*Prasiola*’, including taxa with pseudofilamentous (*Stichococcus*) and pleurococcoid packet-like thalli (*Desmococcus* F. Brand, *Diplosphaera* M.N. Bialosuknia) (Pröschold & Leliaert, 2007; Friedl & Rybalka, 2012). Various morphotypes among different lineages of algae are not the result of their multiple origins, but result from tiny morphological changes that dramatically influence their gross morphology.

## Supplementary information

The following supplemental material is available for this article, accessible via the Supplementary Content tab on the article’s online page at http://dx.doi.org/10.1080/09670262.2014.949308

**Supplementary table 1**. Information for strains of *Klebsormidium* and *Interfilum* used in the study.

## Supplementary Material

Supplementary material
