# Supplementary material for: Morphology and ultrastructure of Interfilum and Klebsormidium (Klebsormidiales, Streptophyta) with special reference to cell division and thallus formation
Source: Eur J Phycol. 2014 Oct 13;49(4):395–412. doi: 10.1080/09670262.2014.949308 (PMC4618308; doi:10.1080/09670262.2014.949308)
Supplement: Supplementary material [file tejp_a_949308_sm6286.doc]

**Supplementary Table 1**

Information for strains of *Klebsormidium* and *Interfilum* used in the study.

| Number of strain in culture collection | Strain label | Identification | Origin | Phylogenetic data | | |
| --- | --- | --- | --- | --- | --- | --- |
|  |  |  |  | Clade | | References |
| SAG 2102 |  | *Interfilum massjukiae* | Karadag Nature Reserve, Crimea, Ukraine; cracks and surface of pyroclastic outcrops | A | Mikhailyuk et al., 2008; Rindi et al., 2011 | |
| SAG 338.1 |  | *Interfilum paradoxum* | Epping, near London, U.K.; soil from beech forest | A | Mikhailyuk et al., 2008; Rindi et al., 2011 | |
| SAG 2100 |  | *Interfilum terricola* | Haute Ardenne, Belgium; soil from oak tree forest | A | Mikhailyuk et al., 2008' Rindi et al., 2011 | |
| SAG2101 |  | *Interfilum* sp. | Granite-Steppe Pobuzhzhia Regional Landscape Park, Ukraine; in cracks of granite outcrop | A | Mikhailyuk et al., 2008; Rindi et al., 2011 | |
| SAG 2147 |  | *Interfilum* sp. | Czech Republic; from soil | A | Mikhailyuk et al., 2008; Rindi et al., 2011 | |
| SAG 36.88 |  | *Interfilum* sp. | Mount Tekoa, New Zealand; from soil | A | Mikhailyuk et al., 2008; Rindi et al., 2011 | |
| SAG 5.96 |  | *Klebsormidium bilatum* | Poppel, Belgium; bank at a brooklet | D | Sluiman et al., 2008; Rindi et al., 2011 | |
| SAG 37.86 |  | *Klebsormidium crenulatum* | Bressanone, Italy; from soil | F | Rindi et al., 2011 | |
| SAG 12.91 |  | *Klebsormidium flaccidum* | Sojovice Reservoir, Czech Republic | B/C | Mikhailyuk et al., 2008 | |
|  | Biof4 | *Klebsormidium* cf. *flaccidum* | Massandra, Crimea, Ukraine; artificial stony substratum | B/C | Rindi et al., 2011 | |
|  | TR 44 | *Klebsormidium* cf. *dissectum* | Boguslav district, Kyiv oblast, Ukraine; granite outcrop | B/C | Rindi et al., 2011 | |
| CCAP 335.12 |  | *Klebsormidium fluitans* | River Gannel, U.K.; in freshwater | E | Rindi et al., 2011 | |
| CCAP 335.17 |  | *Klebsormidium subtile* | River Gannel, U.K.; in freshwater | E | Rindi et al., 2011 | |
| SAG 2107 |  | *Klebsormidium* sp. | Unknown | E | Rindi et al., 2011 | |
| SAG 2108 |  | *Klebsormidium* sp. | Unknown | E | Rindi et al., 2011 | |
|  | TR 18 | *Klebsormidium* sp. | Australia; from soil | E | Rindi et al., 2011 | |
| ACKU 379 | TR 24 | *Klebsormidium* sp. | Granite-Steppe Pobuzhzhia Regional Landscape Park, Mykolaiv district; Ukraine, granite outcrops | B/C | Rindi et al., 2011 | |
| ACKU 801 | TR 42 | *Klebsormidium* sp. | Kyiv oblast Boguslav district, Ukraine; granite outcrop | B/C | Rindi et al., 2011 | |
|  | 14613.5e | *Klebsormidium* sp. | Koebos, South Africa, soil crust | G | Rindi et al., 2011 | |
|  | 14621.6 | *Klebsormidium* sp. | Rocherpan Nature Reserve, Western Cape, South Africa; soil crust | G | Rindi et al., 2011 | |
|  | KUE1 | *Klebsormidium* cf. *flaccidum* | Kühtai, Limnological Station Gossenköllsee, University Innsbruck, Austria, 2435 m a.s.l., soil crust under *Festuca rubra* | B/C | Karsten et al., 2013 | |
| ASIB V100 |  | *Klebsormidium* *flaccidum* | Pitschberg, St. Ulrich in Gröden, South Tyrol Austria, 2.363 m a.s.l., soil crust under *Festuca rubra* | B/C | Original data (accession number HG973008) | |
|  | PIT1 | *Klebsormidium* cf. *bilatum* | Pitz Valley Glacier, Austria, 2843 m a.s.l., soil crust on rocks | D | Original data (accession number HG973012) | |
| SAG 2415 | AH1 | *Klebsormidium crenulatum* | Schönwieskopf, Obergurgl, Austria, 2.350 m a.s.l., alpine soil crust | F | Kaplan et al., 2012 | |
| SAG 2416 | AH2 | *Klebsormidium dissectum* | Schönwieskopf, Obergurgl, Austria, 2.350 m a.s.l., alpine soil crust | E | Karsten & Holzinger, 2012 | |
| SAG 2417 | BOT2 | *Klebsormidium nitens* | Innsbruck, botanical garden, Austria, 550 m a.s.l., concrete panels | E | Kaplan et al., 2012 | |
|  | BOT3 | *Klebsormidium* cf. *fluitans* | Innsbruck, botanical garden, Austria, 550 m a.s.l., concrete panels | E | Kitzing et al., 2014 | |
|  | BRE | *Klebsormidium* cf. *subtile* | Bremen, Germany, 11 m a.s.l., soil crust, rain water discharge | E | Original data (accession number HG973009) | |
|  | HOH2 | *Klebsormidium* cf. *subtile* | Hohenzollernhaus, Radurschl Valley, Austria, 2207 m a.s.l., soil crust | E | Original data (accession number HG973011) | |
|  | STR1 | *Klebsormidium* cf. *nitens* | Strassberg Hut, Austria, 1280 m a.s.l soil crust in pine forest | E | Original data (accession number HG973010) | |
